# 

**Published:** 1802-09-01

**Authors:** 


### To CORRESPONDENTS.

*Communications are received from Messrs. Davis, Chamberlaine, Luscombe, Bellamy, Shorter, W. P., A Magistrate, Brutus, and N. G.*

*Those Gentlemen who have sent us Criticisms on Dr. Pearson's Evidence, and whose Papers do not appear in this Number, are informed that the language they have employed is too violent for our Journal. If they should not be satisfied with the moderate Paper which we have inserted, we request that any further Observations from them or others may be written in language not inconsistent with a spirit of liberal inquiry.*

*We have received several Answers to Mr. Chamberlaine, on the new Medicine Act, and we hope that the Authors are correct, and that Mr. Chamberlaine has been unnecessarily alarmed. But knowing as we do, that Magistrates are generally obliged to inflict penalties according to the* Letter *and not according to the* Spirit *of an Act, we are inclined to take Mr. Chamberberlaine's side of the argument. As, however, some opinions of high legal authority are shortly expected to be given, respecting particular points of this Act, we think it prudent not to give place to further animadversions till those opinions shall be before the Public.*

